# Case report: unusual posteromedial capsular lesion with posterior lateral meniscus root tear in two patients with constitutional genu recurvatum presenting after an acute ACL injury

**DOI:** 10.1186/s40634-023-00684-y

**Published:** 2023-12-13

**Authors:** Giulio Vittone, Caroline Mouton, Jérôme Valcarenghi, Jérémie Dor, Romain Seil

**Affiliations:** 1 Department of Orthopaedic Surgery, Centre Hospitalier Luxembourg – Clinique d’Eich, Clinique d’Eich 78 Rue d’Eich, L-1460 Luxembourg, Luxembourg; 2 Department of Medical and Surgical Specialties, Radiological Sciences and Public Health, University of Brescia, Brescia, Italy; 3 Sports Medicine and Science (LIROMS), Luxembourg Institute of Research in Orthopaedics, Luxembourg, Luxembourg; 4 Department of Orthopaedic Surgery, Centre Hospitalier Universitaire d’ambroise Paré, Mons, Belgium; 5 Orthopaedics, Sports Medicine and Digital Methods (HOSD), Human Motion, Luxembourg Institute of Health, Luxembourg, Luxembourg

**Keywords:** Anterior cruciate ligament reconstruction, Medial meniscus, Ramp lesion, Posteromedial capsule

## Abstract

Ramp lesions of the medial meniscus and posterior lateral meniscus root tears (LMPRT) can be present simultaneously in up to 8% of patients undergoing anterior cruciate ligament (ACL) reconstruction. The prevalence of these complex and highly unstable meniscal tears increases exponentially with the severity of the injury. The posteromedial capsule (PMC) has often been disregarded in the past when discussing ligamentous and meniscal injuries, but the recent interest in ramp lesions has drawn surgeons’ attention to the posteromedial structures of the ACL injured knee. While the meniscocapsular junction is commonly repaired in unstable ramp lesions, in the current literature there is no report regarding proximal PMC lesions, be they in isolation or associated with complex meniscal injuries.

We report here two cases of proximal posteromedial capsular lesions associated with medial meniscus instability and posterior lateral root tears after ACL injury. The first case involves a meniscus ramp lesion associated with a proximal PMC tear and a posteromedial fluid collection in the muscle plane on magnetic resonance in a 22-year-old male soccer player. The second case involves a 21-year-old female soccer player who presented with a PMC lesion after hyperextension/valgus knee injury. The capsular lesions were repaired to restore capsular tension and improve medial meniscus posterior horn stability.

## Introduction

Ramp lesions of the medial meniscus and lateral meniscus posterior root tears (LMPRT) can be present simultaneously in up to 8% of patients undergoing anterior cruciate ligament (ACL) reconstruction [18]. Ramp lesions are longitudinal tears of the meniscotibial and/or meniscocapsular attachments of the posterior horn of the medial meniscus (PHMM) and can be present in up to 40% of ACL ruptures [14, 25]. Ramp lesions lead to abnormal meniscal and tibiofemoral laxity, predisposing the knee to meniscal and articular damage [14, 26]. They are commonly repaired to help restore anterior tibial translation (ATT) and reduce pivot shift [10, 26]. Similarly to ramp lesions, lateral meniscus posterior root tears (LMPRT) are usually traumatic injuries that lead to an increase in meniscal extrusion, ATT and pivot shift [11, 28]. Repair of LMPRT is suggested to lower extrusion and help prevent early osteoarthritis onset [16].

Recent interest in medial meniscus ramp lesions has shed light on the posteromedial structures of the knee and their role in meniscal stability. The posteromedial capsule (PMC) is one of the key elements in the posterior aspect of the knee. It plays a pivotal role in controlling valgus, internal rotation, and posterior drawer in extension [22]. On its tibial extremity, the PMC has also shown an important role as a stabilizing structure of the PHMM through the meniscocapsular ligament [10]. However, on its femoral extremity, little is known regarding the anatomy of the PMC, its biomechanical influence and the treatment of PMC injuries. While the association of ramp lesions and PLRMT in ACL injuries has been previously described [18, 19], to the best of the authors’ knowledge there are no reports regarding proximal posteromedial capsular injuries. Tears in the proximal PMC may contribute to instability in these complex knee injuries and careful consideration may therefore be given to a possible concomitant capsular repair.

In our surgical practice, two rare cases of proximal posteromedial capsular tears associated with LMPRT and ramp lesions after ACL injury were encountered. The aim of this manuscript is to discuss these clinical cases, their surgical management and the results of a short term follow up.

## Case report—patient 1

### Clinical history and examination

**Fig. 1:**
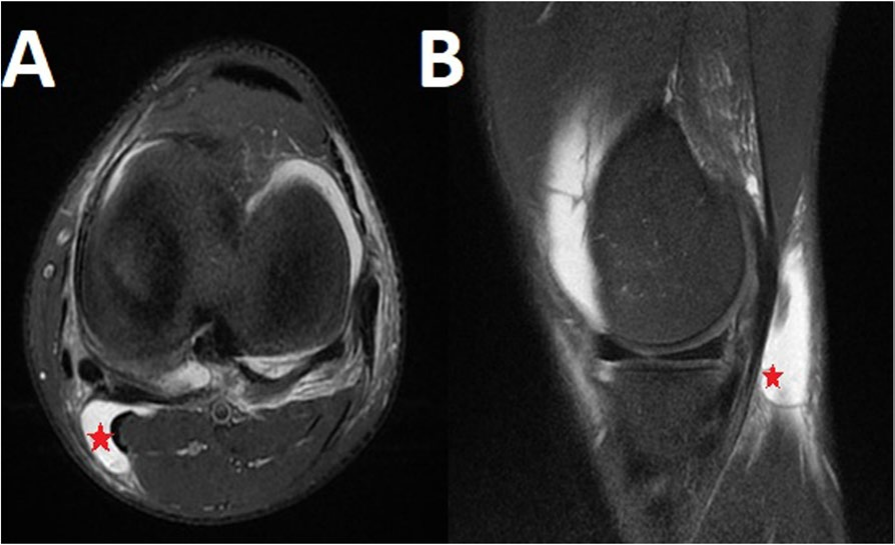
MRI of *case 1*. Left knee axial view (**A**) and coronal view (**B**). These MRI slices show the hyperintensity signal indicative of a fluid collection in the posteromedial muscle plane (red star). The direct arm of the semimembranosus tendon appears intact

A 22 year old male football player presented at the emergency department with pain and joint effusion after a non-contact hyperextension/valgus injury to the left knee during a football match. Clinical history revealed a previous ACL injury followed by reconstruction on the contralateral side, 3 years prior. Clinical examination of the left knee revealed a positive Lachman test (grade II laxity, soft end point) and a grade 2 + pivot-shift test. Range of motion (ROM) evaluation showed a hyperextension of 10° of the left knee (contralateral side: 5°) with full flexion. Dial test was negative, no concomitant varus/valgus instability could be found. Quadrant and McMurray tests were negative. Radiographs of the left knee were negative for bony fractures or abnormalities. The posterior tibial slope measured on lateral X-rays was 10.5°. MRI confirmed ACL rupture and revealed the presence of a lateral meniscus tear and perimeniscal hyperintensity signal behind the PHMM on T2 sequence indicative of a ramp lesion [4, 13]. MRI also showed an uncommon fluid collection in the posteromedial muscle plane (Fig. 1), surrounding the distal semimembranosus (SM) tendon which appeared continuous and homogeneously hypointense in its distal direct and anterior arms. After clinical evaluation the patient was considered eligible for arthroscopic ACL reconstruction with lateral extra-articular tenodesis and concomitant meniscal repair surgery.

### Diagnostic arthroscopy

**Fig. 2:**
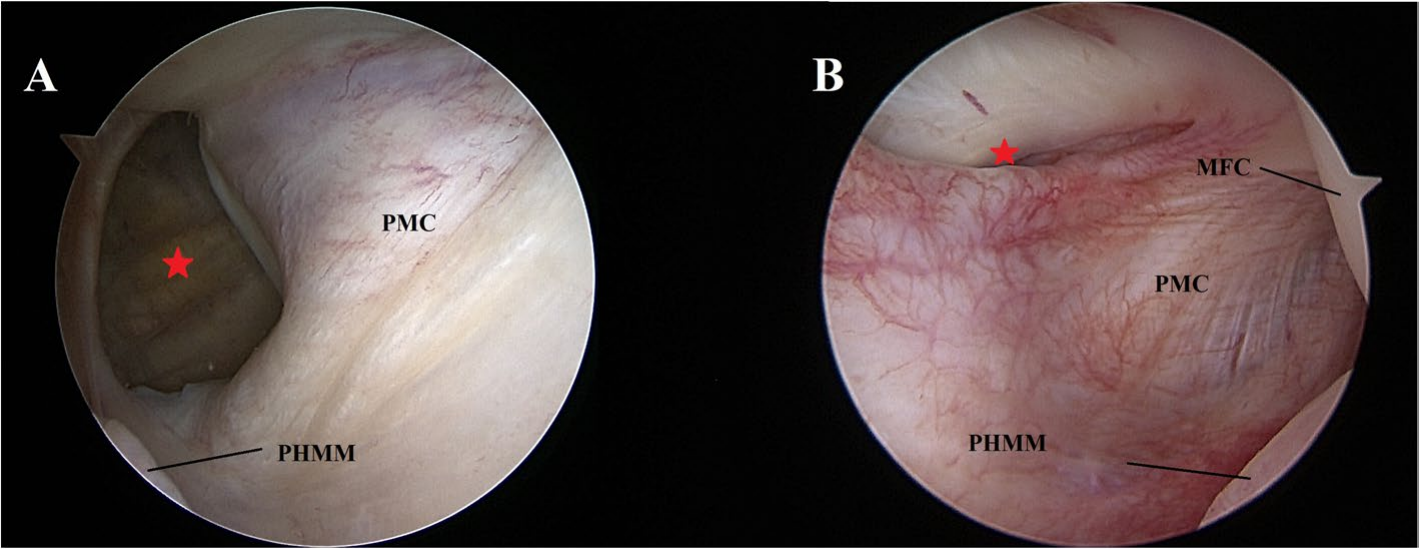
Trans-notch view of the posteromedial compartment during arthroscopy: *case 1* (**A**), *case 2* (**B**). Visualization of the posteromedial compartment reveal the presence of posteromedial capsular lesions (red stars). The lesion depicted in *case 1* (**A**) is more evident, while in *case 2* (**B**) a synovial fold covers the tear*.* MFC: medial femoral condyle; PMC: Posteromedial capsule; MM: medial meniscus

**Fig. 3:**
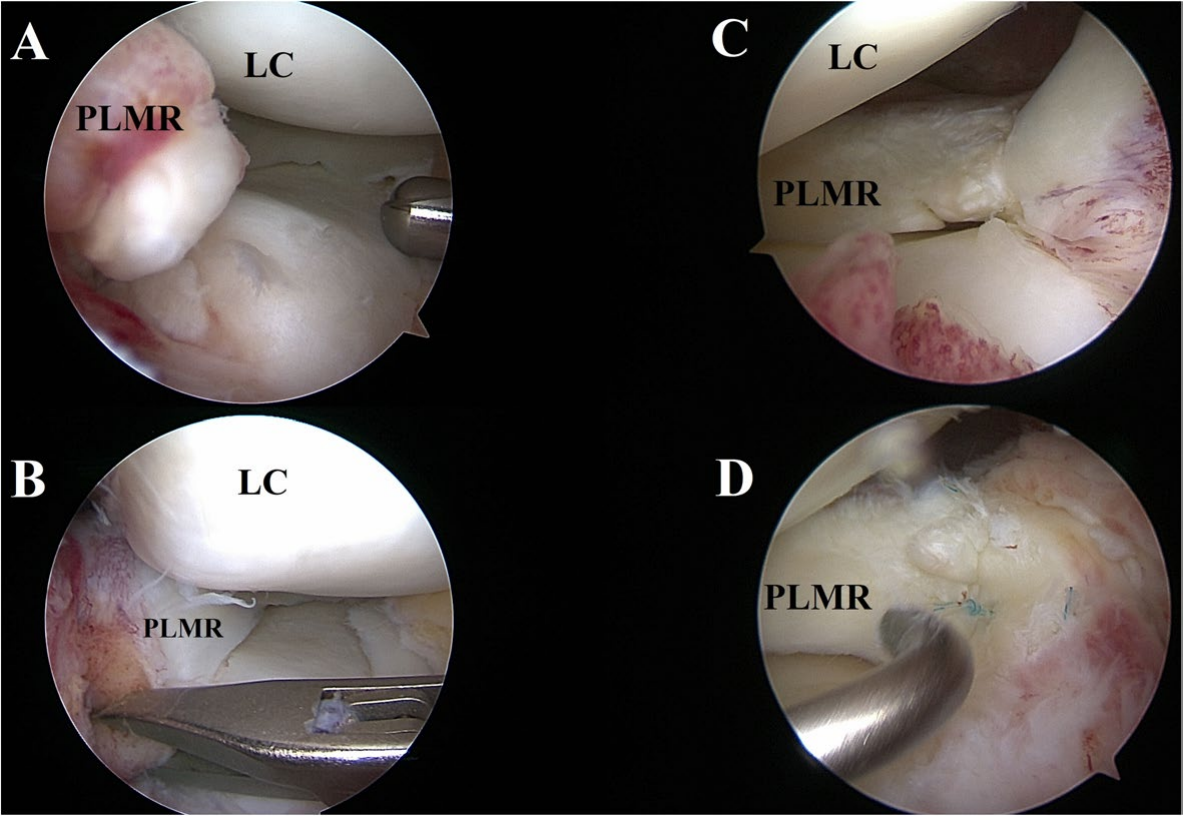
Arthroscopic view of the posterior lateral root in *case 1* (**A**, **B**) and *case 2* (**B**, **C**). In *case 1* a type II posterior lateral root tear is shown before (**A**) and after reduction with an arthroscopic forceps (**B**). In *case 2* a type II posterior lateral root tear is shown before (**C**) and after repair with all-inside sutures (**D**). LC: Lateral condyle LMPR: lateral meniscus posterior root

Surgical treatment was carried out at 4 weeks from the initial traumatic event after the patient had recovered a pain free knee. Standard diagnostic arthroscopy confirmed the presence of a complete ACL rupture and revealed a complex corollary of associated soft tissue injuries. On the medial femoral condyle, a Crevice sign was present (Video 1). The latter has been previously described as a pathognomonic sign of an unstable posterior medial meniscal tear in ACL-deficient knees [20]. The transnotch view of the PM compartment then confirmed the presence of an unstable ramp lesion with a complete tear in the red zone (type IV according to Thaunat et al. [27]) and revealed an uncommon associated proximal PM capsular lesion (Fig. 2A; Video 1). In the lateral compartment, a type II lateral meniscus posterior root tear (Fig. 3A, B), coupled with a radial tear of the midbody, was present.

To determine and classify the severity of the injury, the ACL injury severity scale (ACLISS) [23] was used. This scoring system combines MRI findings with intra-articular diagnostics to identify associated injuries to the surrounding soft tissues and bone in the event of an ACL injury. An ACLISS grade III injury (Table 1) was confirmed in patient 1, indicating high severity.

**Table 1:** Patient 1—Anterior cruciate ligament injury severity scale [23] (ACLISS): grade III

Category	Tibiofemoral compartment				Score per category
	Medial	Score	Lateral	Score	
Ligament	Grade 1 MCL injury	1	/	/	1/1
Meniscus	Major unstable MM tear	2	Major unstable LM tear	2	4/4
Bone	Bone bruise of TP	1	Bone bruise of TP AND FC	2	
	/	/	No associated fracture	0	3/5
Cartilage	No grade 3 or 4 lesion	0	No grade 3 or 4 lesion	0	0/2
Score per compartment		4/6		4/6	Total: 8/12 **Grade: III**^**a**^

*FC* Femoral Condyle, *LM* Lateral Meniscus, *MCL* Medial Collateral Ligament, *MM* Medial Meniscus, *TP* Tibial plateau

^a^ACLISS grade I score: < 4 (Mild severity); ACLISS grade II score: between ≥ 4 and ≤ 7 (moderate severity); ACLISS grade III score: > 7 (high severity)

### Operative treatment

**Fig. 4:**
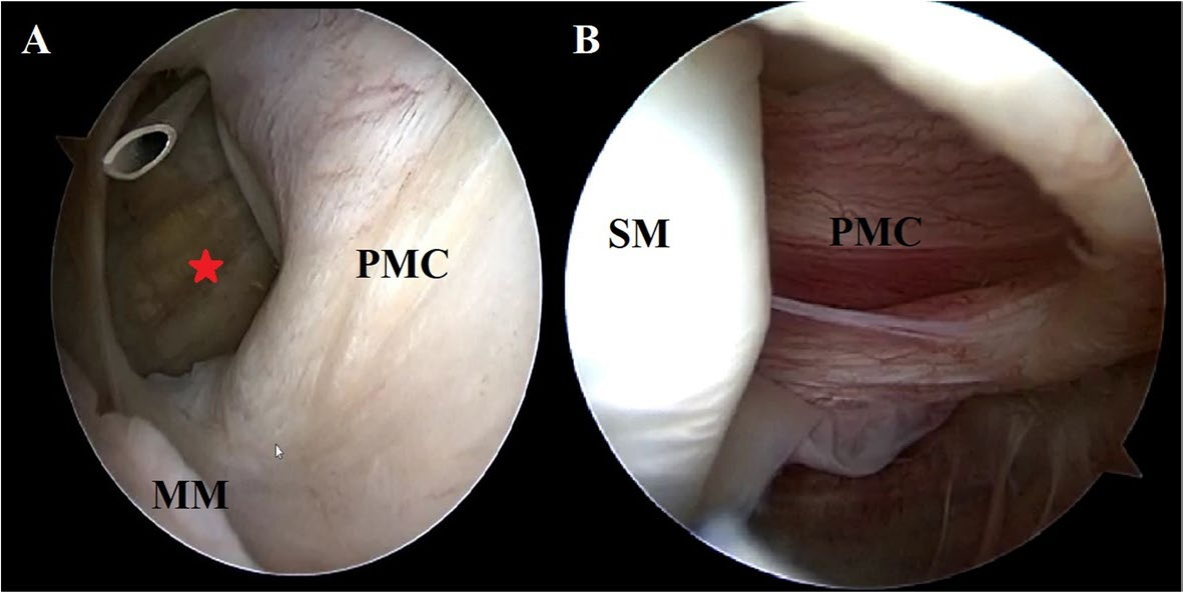
Trans-notch view of the posteromedial capsular lesion *case 1* (**A**). The needle indicates the normal positioning of the posteromedial viewing portal described by Siboni et al. [24]. Once established this portal allowed to explore the extra-articular component of the tear (**B**) and revealed a partial tear of the semimembranosus (SM) tendon. From this perspective it is possible to appreciate the relationship between the SM and the posteromedial capsule (PMC). PMC: Posteromedial capsule; SM: semimembranosus; MM: medial meniscus

**Fig. 5:**
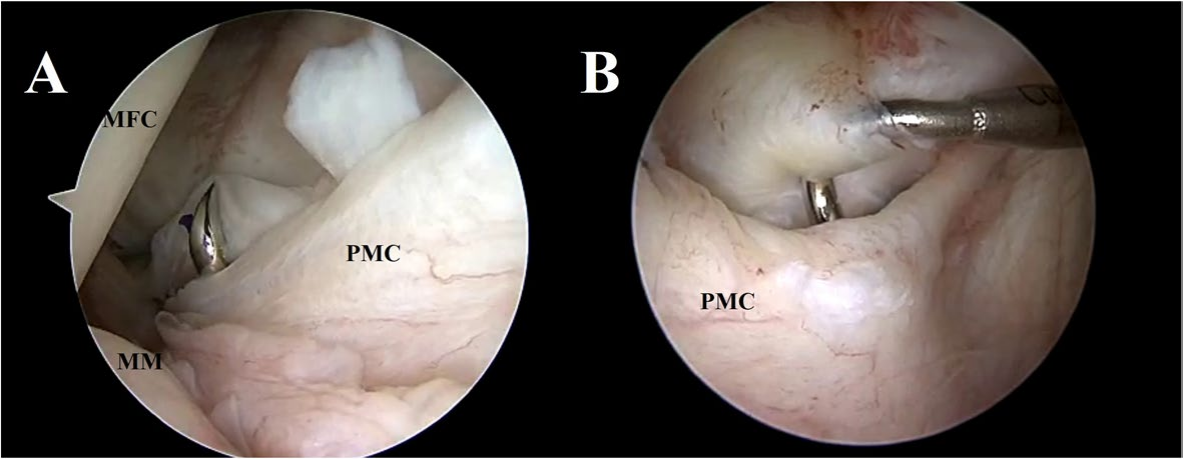
Trans-notch view—*case 1* (**A**)—*case 2* (**B**). Suture hook repair of the posteromedial capsular lesions. MFC: medial femoral condyle; PMC: Posteromedial capsule; MM: medial meniscus

**Fig. 6:**
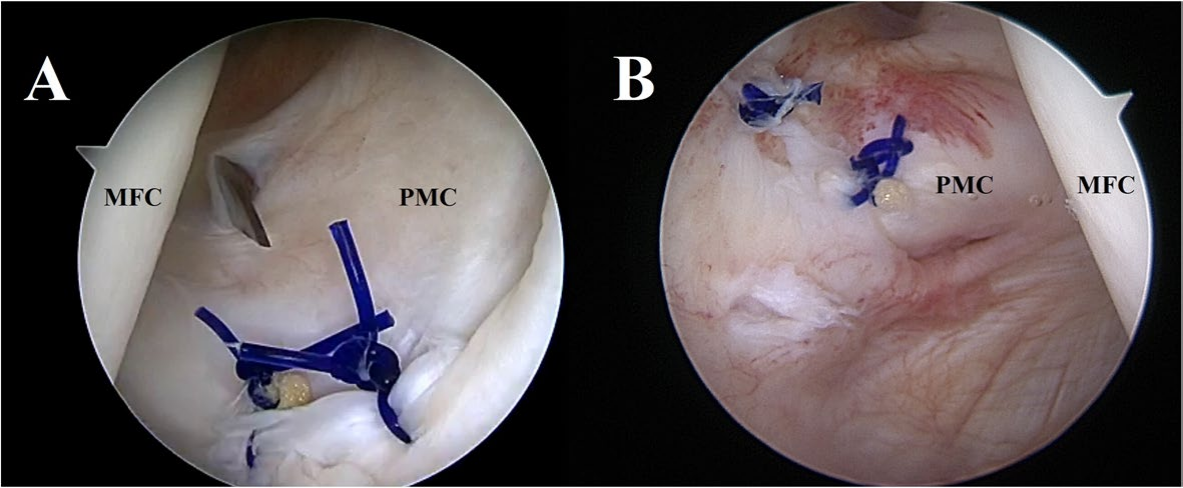
Trans-notch view—*case 1* (**A**) – *case 2* (**B**). The images show the appearance of the posteromedial capsule after repair. The capsular tension is restored after repair of the proximal capsular lesion and the synovial fold described by Siboni et al. [24] becomes visible again. The blade in fig. A indicates the position of the new viewing portal established to perform a 2-posteromedial portal ramp repair in *case 1*. MFC: medial femoral condyle; PMC: Posteromedial capsule

**Fig. 7:**
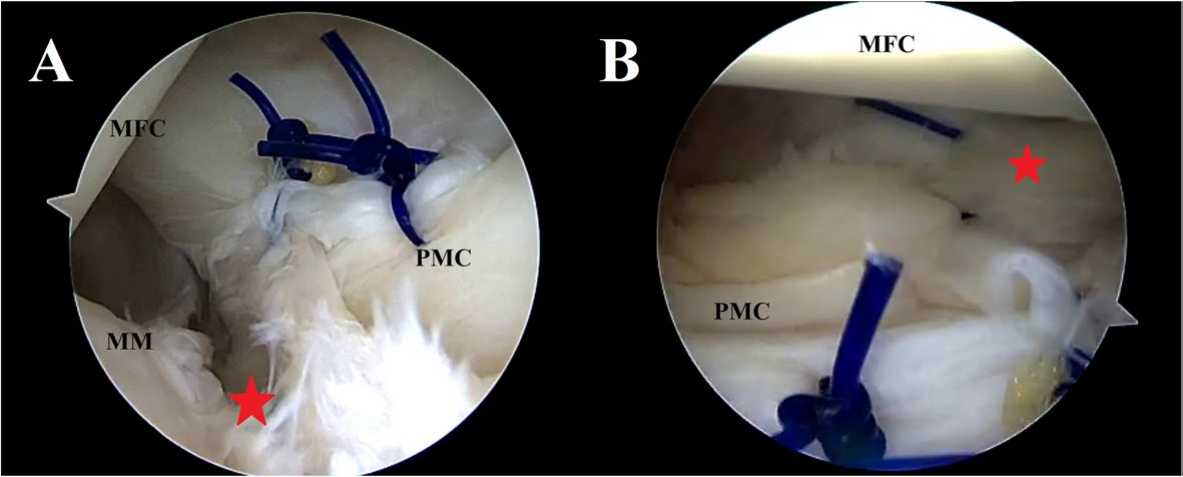
**A** Trans-notch view *case 1*. This image shows the ramp lesion (red star) after repair of the proximal capsular lesion. **B** Posteromedial view *case 1*. Final result of the capsular repair and the ramp repair (red star). MFC: medial femoral condyle; PMC: Posteromedial capsule; MM: medial meniscus

An attempt to repair the ramp lesion on the PHMM was carried out using a 2-portal posteromedial approach [24]. The PM viewing portal position corresponded with the proximal capsular lesion previously observed (Fig. 4A). Placing the scope through the lesion allowed a clear visualization of the associated injuries in the extra-articular compartment, revealing a partial tear of the capsular branch of the distal SM tendon (Fig. 4B; Video 1). The loss of tension resulting from the presence of the PMC lesion hindered the stability of the meniscocapsular junction and did not allow for adequate suture hook repair of the ramp lesion. It was thus decided to repair the proximal capsular lesion before proceeding with the standard ramp repair. A trans-notch view was used while instruments were passed through the PM instrumental portal (Fig. 5A). The instrument of choice for the repair was a 90° left curved suture hook loaded with a PDS 1. Two stitches were placed starting from the proximal pole of the tear. Repair of the capsular lesion restored tension on the PM capsular complex (Fig. 6A; Video 1) allowing the creation of a new posteromedial viewing portal and ramp repair to be carried out in the standard fashion as described by Siboni et al. [24] (Fig. 7A, B). Assessment of the PMC tension during repetitive flexion–extension movements was performed after repair to evaluate the stability of the construct.

Repair of the lateral meniscus root tear was then carried out using a transtibial double-tunnel pullout technique, while the radial tear was repaired using three out-in sutures. After meniscal repair, ACL reconstruction was carried out using quadriceps autograft with bone-block and double interference screw fixation, followed by a lateral extra-articular tenodesis with a modified Lemaire technique.

### Post-operative rehabilitation and follow-up

After the operation, the knee was put in an extension brace for 6 weeks, without weight-bearing, to allow for healing of the ramp lesion and the lateral meniscus root tear. Daily removal of the brace and passive mobilization without weight-bearing was allowed up to 90° of flexion. Isometric quadriceps contraction and abduction/adduction exercises were encouraged. The brace was removed at 7 weeks and the patient was allowed to start progressive gait and ROM rehabilitation. At 6 months follow-up, upon clinical examination, the knee was stable without pain or joint effusion. The patient had returned to walking without symptoms or limping and had regained almost full range of motion (ROM 0–0-120°). The patient was allowed progressive return to running and non-contact sport training, subsequent evaluation was scheduled at 8 months post-op.

## Case report—patient 2

### Clinical history and examination

A 21-year-old female football player presented at the emergency department with pain and joint effusion after a contact hyperextension/valgus injury to the right knee during a football match. Clinical examination of the right knee revealed a positive Lachman test (grade II laxity, soft end point) and a grade 2 + pivot-shift test. Range of motion (ROM) evaluation revealed significant limitation (0–10-80°) in the presence of important joint effusion. Analysis of the contralateral knee showed constitutional hyperlaxity with a genu recurvatum between 5°-10°. A concomitant valgus instability in extension with pain at palpation on the femoral insertion of the medial collateral ligament (MCL) could be found. Radiographs of the left knee were negative for bony fractures or abnormalities. The posterior tibial slope measured on lateral X-rays was 9.5°. MRI confirmed ACL and MCL rupture and revealed the presence of a lateral meniscus tear. The patient was considered eligible for arthroscopic ACL reconstruction, lateral extra-articular tenodesis and concomitant meniscal repair surgery, after a 6 weeks conservative treatment for the MCL injury and physiotherapy treatment to regain full ROM and a pain free knee.

### Diagnostic arthroscopy and operative treatment

Surgical treatment was carried out at 4 months from the initial traumatic event. Standard diagnostic arthroscopy confirmed the presence of a complete ACL rupture, a type II posterior lateral root tear (Fig. 3 C, D) and a grade II trochlear chondropathy. The transnotch view of the PM compartment confirmed the absence of a ramp lesion and revealed the presence of a proximal PM capsular lesion (Video 1; Fig. 2B). The lesion produced a loss of tension in the PMC observed also through a posteromedial view (Video 1). It was thus decided to repair the proximal capsular lesion before proceeding with the LMPRT repair. A trans-notch view was used while instruments were passed through the PM instrumental portal (Fig. 5B). The instrument of choice for the repair was a 90° right curved suture hook loaded with a PDS 1. Two stitches were placed starting from the proximal pole of the tear (Fig. 6B). Repair of the capsular lesion restored tension on the PM capsular complex. Assessment of the PMC tension during repetitive flexion–extension movements was performed after repair to evaluate the stability of the construct.

Repair of the LMPRT was then carried out using 2 all-inside suture devices. After meniscal repair, ACL reconstruction was carried out using quadriceps autograft with bone-block and double interference screw fixation, followed by a lateral extra-articular tenodesis with a modified Lemaire technique. After combining the MRI findings with the intra-articular diagnostics a combined ACLISS [23] grade II (Table 2) injury was confirmed, indicating a moderate severity of the injury.

**Table 2:** Patient 2 – Anterior cruciate ligament injury severity scale [23] (ACLISS): grade II

Category	Tibiofemoral compartment				Score per category
	Medial	Score	Lateral	Score	
Ligament	Grade 2 MCL injury	1	/	/	1/1
Meniscus	Major unstable MM tear	2	Major unstable LM tear	2	4/4
Bone	No bone bruise	0	Bone bruise of TP AND FC	2	
	/	/	No associated fracture	0	2/5
Cartilage	No grade 3 or 4 lesion	0	No grade 3 or 4 lesion	0	0/2
Score per compartment		3/6		4/6	Total: 7/12 **Grade: II**^**a**^

*FC* Femoral Condyle, *LM* Lateral Meniscus, *MCL* Medial Collateral Ligament, *MM* Medial Meniscus, *TP* Tibial plateau

^a^ACLISS grade I score: < 4 (Mild severity); ACLISS grade II score: between ≥ 4 and ≤ 7 (moderate severity); ACLISS grade III score: > 7 (high severity)

### Post-operative rehabilitation and follow-up

The post-operative rehabilitation protocol was analogous to the one previously described. At 4 months follow-up, upon clinical examination, the knee was stable without pain or joint effusion. The patient had returned to walking without symptoms or limping and had regained full range of motion (ROM 0–0-135°). The patient was allowed to start progressive strength training, subsequent evaluation was scheduled at 6 months post-op.

## Discussion

In this paper, an uncommon association of proximal posteromedial capsular lesions and posterior lateral root tears after ACL injury has been described in 2 patients presenting with constitutional genu recurvatum.

The evergrowing body of literature has shown us how ACL injuries are seldom isolated events [12, 18, 23]. A significant amount of structural damage to other intra and extra-articular structures is present in nearly 65% of patients undergoing ACL reconstruction [23]. Significant progress in recent years has been made in identifying meniscal and ligamentous injuries that were often overlooked in the past, but little consideration has been given to the role of the knee capsule. However, recent interest in medial meniscus ramp lesions however, has shed light on the posteromedial structures of the knee and their role in meniscal stability. The importance of the meniscocapsular attachment in stabilizing the PHMM has been firmly established [10, 26] and when taking this into account, it is only natural to suppose that proximal PMC tears, if left unattended, could result in insufficient capsular tension and therefore leave residual instability. A fitting comparison for this injury might be the humeral avulsion of the glenohumeral ligament (HAGL) in the shoulder, in which a lesion on the opposite side of the labrum produces significant effects in terms of joint stability [9]. Furthermore, the first patient presented a bipolar lesion consisting of two tears: a ramp tear and one in the proximal PMC. In the shoulder bipolar avulsions of the inferior glenohumeral ligament, although rare, have been previously described [7, 29]. These lesions result from a combination of HAGL and a “Bankart lesion”. Repair of bipolar injuries in the shoulder involves restoration of both the glenoid and humeral attachments, as failure to address both sides has been reported to result in a high rate of recurrent anterior instability and isolated Bankart repair failure [2]. Drawing a lesson from shoulder surgery we decided to perform suture hook repair of the proximal PMC lesions, believing it would help restore the meniscus function, and protect the ramp repair.

The presence of an unstable tear of the lateral meniscus such as a LMPRT has been associated with an increased risk of injury to other secondary knee stabilizers [12, 23]. Seil et al. [23] recently developed a tool for the evaluation of structural damage in the ACL-injured knee. The authors reported that while overall tissue damage is predominant in the lateral tibiofemoral compartment, the proportional involvement of the medial compartment increases with the severity grade of the injury.

Interestingly their findings suggest that, similarly to medial compartment involvement, the prevalence of major unstable lateral meniscus tears scales with the severity of the ACL injury, going from 12% in low grade ones to 81% in the most severe cases. LMPRTs may therefore be considered as an alarm bell and should prompt an even more accurate analysis of the medial and posteromedial aspect of the knee.

Genu recurvatum has been shown as a potential risk factor for ACL injury [6]. Both of our patients presented with constitutional genu recurvatum, this may have facilitated hyperextension trauma. In a recent biomechanical analysis Noyes et al. [21] have shown that the posteromedial and posterolateral capsular structures provided the major resisting moment to prevent knee hyperextension. The role of the posterior capsule in preventing hyperextension, led us to think that the PMC tear might have contributed to the increase in hyperextension seen acutely in the first patient. This theory however could not be confirmed in the second patient where ROM was limited due to significant joint effusion. Noyes et al. [21] also emphasized the possible role and importance of concomitant posterior capsular structures’ repair in ligamentous injuries. Based on previous biomechanical analyses it has been hypothesized that a functional capsule could contribute to resisting hyperextension forces absorbing part of the strain that’s placed on the ACL reconstruction graft [3, 21].

An interesting finding in the first patient was the presence of a posteromedial fluid collection in the muscular plane, clearly identifiable on MRI, and confirmed under arthroscopic examination. Passing the arthroscope through the PMC lesion allowed us to identify a partial tear of the distal SM tendon. The existing body of literature concerning injuries to the semimembranosus tendon is notably sparse, predominantly comprising isolated case reports [1, 5, 15]. These reports primarily focus on distal avulsion injuries or instances of complete tendon ruptures. The mechanism of injury however seems consistent between the different reports, indicating hyperextension as the mechanical cause, similar to our case. The relationship between the PHMM and the distal SM has been extensively investigated [5, 8]. Anatomical evaluations demonstrated that the distal semimembranosus complex is participating in the posterior translation of the meniscus during knee flexion thanks to its capsular branch, inserted behind the medial meniscus [8]. In a previous anatomical study, Laprade et al. [17] described the presence of a physiologic posteromedial capsular defect or thinning, distal to the medial head of the gastrocnemius attachment and proximal to the direct arm attachment of the SM. This physiologic posteromedial defect could represent a point of weakness and favor the development of PMC lesions in this zone due to SM traction.

Although the capsular tear of the first patient was blatant, less evident posteromedial capsular injuries like the one described in the second patient have occasionally been observed in our practice, especially during acute ACL reconstructions, before scarring and remodeling takes place. The incidence of these lesions is yet unknown, as there is a lack of literature and knowledge on the subject. In the future, prospective studies might be useful to determine the prevalence of these injuries and to evaluate their impact on knee stability. This would also help to gain insight in the possible role of capsular repair procedures.

We believe this report strengthens the recommendation for routine diagnostic arthroscopy of the posteromedial compartment in ACL reconstructions, especially after hyperextension injuries and in the presence of lateral root tears. Systematic evaluation of the PMC can help identify these lesions that might contribute to residual posteromedial instability after ACL reconstruction. Furthermore, this highlights the importance and role of the capsule in ramp lesions, leading us to consider the PMC and PHMM as a single functional unit in stabilizing the knee. In the event of a posteromedial capsular lesion, repair should be considered as leaving these lesions unattended might result in persistent instability.

## Conclusions

In the current paper, we detailed two clinical cases where patients with constitutional genu recurvatum experienced a rare combination of proximal posteromedial capsular tears alongside lateral meniscus root tears and concurrent medial meniscus instability following ACL injury. It is crucial to emphasize that addressing posteromedial capsular lesions with proactive repair should be considered, as neglecting these issues may lead to persistent meniscal instability. Moreover, our findings underscore the significance of a systematic diagnostic arthroscopy approach, specifically directed towards the posteromedial compartment, when confronted with a lateral posterior meniscus root tear. This approach is pivotal for optimizing clinical management and ensuring favorable patient outcomes.

## Data Availability

Not applicable.

## Abbreviations

LMPRT: Lateral meniscus posterior root tears
ACL: Anterior cruciate ligament
PHMM: Posterior horn of the medial meniscus
ATT: Anterior tibial translation
PMC: Posteromedial capsule
MRI: Magnetic resonance imaging
SM: Semimembranosus
ROM: Range of motion
HAGL: Humeral avulsion of the glenohumeral ligament
MCL: Medial collateral ligament
ACLISS: ACL injury severity scale
